# Accountability in the health system of Tamil Nadu, India: exploring its multiple meanings

**DOI:** 10.1186/s12961-019-0448-8

**Published:** 2019-04-27

**Authors:** Rakhal Gaitonde, V. R. Muraleedharan, Miguel San Sebastian, Anna-Karin Hurtig

**Affiliations:** 1 Department of Public Health and Clinical Medicine, Umea University, Umea, Sweden; 2 Department of Humanities and Social Sciences, Indian Institute of Technology Madras, Chennai, India

**Keywords:** Accountability, Community-based accountability, National Rural Health Mission, Belief structures, Problematisation, Policy implementation

## Abstract

**Background:**

Accountability is increasingly being demanded of public services and is a core aspect of most recent frameworks of health system strengthening. Community-based accountability is an increasingly used strategy, and was a core aspect of India’s flagship National Rural Health Mission (NRHM; 2005–2014). Research on policy implementation has called for policy analysts to go beyond the superficial articulation of a particular policy intervention to study the underlying meaning this has for policy-makers and other actors of the implementation process and to the way in which problems sought to be addressed by the policy have been identified and ‘problematised’.

**Methods:**

This research, focused on state level officials and health NGO leaders, explores the meanings attached to the concept of accountability among a number of key actors during the implementation of the NRHM in the south Indian state of Tamil Nadu. The overall research was guided by an interpretive approach to policy analysis and the problematisation lens. Through in-depth interviews we draw on the interviewees’ perspectives on accountability.

**Results:**

The research identifies three distinct perspectives on accountability among the key actors involved in the implementation of the NRHM. One perspective views accountability as the achievement of pre-set targets, the other as efficiency in achieving these targets, and the final one as a transformative process that equalises power differentials between communities and the public health system. We also present the ways in which these differences in perspectives are associated with different programme designs.

**Conclusions:**

This research underlines the importance of going beyond the statements of policy to exploring the underlying beliefs and perspectives in order to more comprehensively understand the dynamics of policy implementation; it further points to the impacts of these perspectives on the design of initiatives in response to the policy.

## Background

Governments worldwide are under pressure to ensure the provision of basic services to their citizens, with accountability forming a core component of most approaches to strengthening public systems in general and health systems in particular. Accountability is part of many recent frameworks for strengthening health systems and governance [1–3]. Most authors would agree that, at its core, accountability is about ‘answerability’, namely the obligation to inform and explain with transparency, and ‘enforceability’, namely compliance through review and sanctioning mechanisms [4]. While conceptually there seems to be little disagreement over this, there seems to be wide diversity in the way this is translated into practice, as authors over decades have described accountability as being a vague, nebulous or chameleon-like like concept [5–7].

Accountability has also been described as having political, fiscal, administrative, and legal or constitutional aspects, or alternatively as having either vertical (external mechanisms primarily involving citizens and communities) or horizontal (internal mechanisms between different branches of government or through specifically constituted organisations for the same) ‘directions’ [8]. Alternative ‘hybrid’ forms, which invite ‘external’ members into ‘internal’ mechanisms, thus combining aspects of both vertical and horizontal accountability, have also been suggested [9]. Recent conceptualisations have tended to be more community based compared to the older forms centred in bureaucracies [10].

Given varying approaches in practice, it seems to make sense to work towards increased clarity during implementation by answering more specific questions regarding the content of the concept such as who is accountable to whom, for what, by which standards and why? [11]. In addition to these, van Belle has suggested one more dimension – the causal model – which refers to the “*processes and instruments, the contextual conditions and expected outcomes*” of processes related to accountability [7]. In another approach, public health accountability has been described as being used to describe at least four dimensions – as a strategy to ensure the quality of care, as part of community participation, as part global health policy in order to improve ‘donor accountability’, and as framed in ethics and human rights perspectives [7]. These various approaches highlight not only the complexity of the concept but also point to some attempts at increasing clarity in use.

A number of studies have also shown that the health system setting is unique from other public accountability settings. This is especially so given that the health sector is characterised by an “*asymmetry of information and influence among the growing number of health system stakeholders, who have specific interests and different positions of power which may affect policy development*” [12]. Other reviews have pointed out other aspects of this uniqueness, which include the “*size and scope of health care bureaucracies in both the public and private sectors*” with “*significant powers to affect people’s lives and well-being*”, and the fact that healthcare constitutes a “*major budgetary expenditure in all countries, and proper accounting for the use of these funds is a high priority*” [13]. This underlines both the uniqueness and importance of accountability initiatives in the health sector.

Studies of public health systems in low- and middle-income country settings indicate that measures to increase accountability are usually undertaken between actors of very unequal power [8]. A review of 21 projects funded by the World Bank, which explicitly set out to strengthen accountability processes, noted that the focus of the projects was more on quality of services than on their composition, focused more on front-line workers than on higher officials who actually designed the programmes, and were heavily dependent on bureaucracy for their continuation [14]. In a review that focused on patients and clinical settings, it was noted that patients did not have significant opportunities to raise their voice and that, while they were able to make a limited number of choices, this did not have the political impact of making these processes truly participatory [15].

In India, the National Rural Health Mission (NRHM), a flagship health system level intervention at the national level, has brought accountability to the centre stage. Acknowledging the prevalent inequity and the lack of access of quality healthcare to large parts of the population, this mission set out to bring about an “*architectural correction*” of the healthcare system to ensure that all have access to healthcare [16]. The mission spoke of ‘communitisation’ as one of the five core pillars of its approach. Communitisation referred to increasing community ownership of the healthcare system and community-based accountability was a core component. The NRHM introduced a number of interventions at the community level, a few of which, such as the community-based health worker, the village health and sanitation committee, and the process of community-based monitoring and planning (later renamed as Community Action for Health (CAH)), had the enhancement of accountability of health systems to the people as a key feature. They did so by mandating greater community participation and community-based monitoring of services that were guaranteed at public health institutions. In addition, there was a mandate to the various state governments to include non-governmental organisations (NGOs) in the implementation of the various programmes of the NRHM [16]. Despite this mandate in the policy documents, implementation across the country has been patchy, with a number of studies showing the varied interpretation of the concept in practice and diverse pathways of roll-out across the country [17–20]. These studies broadly pointed out the limited way in which the concept of accountability was able to be operationalised, and the limited amount by which the power differential between the system and the community was reduced.

This call for more accountability is further emerging in parallel with institutional transitions in public systems that have changed traditional relationships between states and citizens in complex ways as well as the capacity of the state to respond [21]. This is especially important to study in states like Tamil Nadu, whose public systems, including the public health system, are considered as among the best performers in the country. Furthermore, the patchy nature of implementation of accountability initiatives points to a possibility of different stakeholders holding quite different meanings or perspectives [20]. This underscores the importance of, “*understanding the organizations involved, their ideologies, values and organizational culture*” during the implementation of these processes [14].

The aim of this study was to explore the different ways in which calls for increased accountability as a clear policy directive at the national level were interpreted and acted upon at the state level by different actors in the context of the NRHM in the state of Tamil Nadu.

## Methodology

### The research setting

The Tamil Nadu public systems are remarkable in their relative efficiency compared to those of other Indian states [22, 23]. Similar to the structure in the rest of India, Tamil Nadu has a multi-tiered set-up with a health sub-centre with an auxiliary nurse midwife for every 5000 population, a Primary Health Centre with two doctors at the 30,000 population level and a First Referral Unit or Community Health Centre at the 100,000 population unit – further referral is to the district hospitals and the medical college hospitals in urban centres. Tamil Nadu is the only state in India that has a separate department for public health in charge of the primary care level. This department is further unique in being staffed by doctors trained in public health, unlike the rest of the country where public health training is not mandatory for officers looking after the public health functions. In Tamil Nadu, the state level Department of Public Health and Preventive Medicine is in charge of policy formulation and the subsequent monitoring of implementation. The district level is in charge of the implementation. The hierarchical nature of the Tamil Nadu health system has been described in the literature [22].

The involvement of civil society groups as co-developers and implementers of the programme was due to the spaces created for the NGOs by the NRHM. While the NRHM created the same imperative for accountability for the whole country, it will be important to study how this played out in the unique context of the Tamil Nadu health system.

### The conceptual framework

This research attempts to uncover the underlying beliefs and meanings regarding accountability as they are reflected in the emergent policy and programme design of CAH at the state level. This way of ‘seeing’ policy draws on the interpretive approach of policy analysis represented by authors such as Fischer [24], Yanow [25] and Bacchi [26]. The key insights of this framing include the fact that the choice of issues to focus on, the way they are framed and the solutions offered for their alleviation are not merely technical inputs into policy. Indeed, in each of these steps, the perspectives of those in positions of power and the institutions in which the processes are embedded play an important role in the way in which policy was articulated. It is these beliefs that are in turn translated into programmes and ultimately contribute to the shaping of institutions. The emergent narratives were further analysed along the following sets of dimensions. The first set was defined by Bevan and includes “*who is accountable to whom, for what, by which standards and why?*” [11]. The second set was defined by van Belle and is referred to as the ‘causal model’ [7], which answers the questions – what are the processes and instruments, the contextual conditions and expected outcomes?

### Study design

This study focuses on the policy-making level in the Tamil Nadu health system. We see this as a key level at which reinterpretation and rearticulation of any policy coming from the central government occurs. This process of reinterpretation imbues the policy with the specificities of the state context. This level also crucially constrains the implementation at lower levels. We attempted to understand the various underlying perspectives of the interviewees with reference to the concept of accountability by focusing on which particular policy and programme recommendations they highlighted as representing accountability in practice. We therefore decided to conduct a qualitative study that used in-depth interviews with actors in the process of formulation and implementation of the NRHM at the national and state levels, from both the government and civil society.

### The choice of research participants

We included five government officials (four from the state level and one from the national level) and four civil society representatives (two from the state and two from national level). The main criteria of their choice at the state level was the fact that they were involved in the implementation of the NRHM projects, including those for community-based accountability. Three of the government officials had more than 30 years of experience, while the other two had approximately 15 years. All civil society representatives had over 15 years’ experience. Thus, they all had experienced the various changes in the health system that are being referred to, and some were able to contribute a longer historical perspective of change.

### Data collection

The data for the analysis was collected through in-depth interviews. A common guide with key areas to probe based on the overall objectives of the study was first developed by RG and discussed and finalised with the other authors. Subsequently, RG approached each of those identified during the design process, and held the interviews at locations and times most convenient to the interviewees. The interviews, conducted in English, lasted for approximately an hour, and were recorded with consent. The in-depth interviews covered various areas regarding the community-based accountability processes in the state but focused on how the participants (1) defined what they meant by accountability; (2) described how, in their opinion, an ideally accountable health system would look like; and (3) gave examples of programmes they have been involved in that, in their opinion, had features of or attempted to establish such ideally accountable systems. We included these particular areas for exploration in order to tap into the deeper level understandings of accountability, by engaging with the examples they gave rather than the answers to direct questions. These were then transcribed and the transcription checked against the original recording by RG. Once finalised, these were stored as secure soft copies.

### Data analysis

For the first stage of the analysis we used grounded theory [27] to explore the various meanings that were identified in the interviews. All transcripts were shared with all authors and after multiple rounds of discussion between RG and the other authors, RG proceeded to code the data. After an initial line-by-line open coding, which was close to the data, a set of key categories describing various aspects of the definition of accountability and highlighting various aspects of programmes were identified. Analysing the interviews led to the emergence of a number of specific aspects of the process of implementing the project that were repeatedly highlighted by the participants. These included aspects such as the overall role of the health system; the overall outcomes of programmes aiming to increase system accountability; the role of training or capacity-building of the community in such programmes; the role of community-based monitoring of the availability and quality of entitled services; and the role of external agencies, especially civil society groups, in the implementation of programmes.

In the next step, we analysed each of these specific aspects of the programme through the lens of ‘problematisation’. This involved the identification of the specific meaning that was invoked by the participant that allowed them to portray that particular feature of the programme as exemplifying a high level of accountability. In other words, by studying the proposed intervention, we were able to discern how the issue was being thought about [26]. Thus, when a participant identifies accountability solely as the achievement of expert-set numerical targets, we asked what the underlying meaning or perspective of accountability would be that enables someone to posit the achievement of numerical targets as being accountable.

Further, we read closely within the material describing specific aspects of the programme to discern any contradictions between participants or contrasting assumptions. We then repeated the exercise for each of the aspects of the programmes described above. After repeated iterations of these steps, we were able collect these assumptions or perspectives into three emergent groups of policy framings, which are described in detail in the results section.

## Ethics

This study received ethics clearance from the Institutional Scientific and Ethics committee of the Society for Community Health Awareness Research and Action – School of Public Health Equity and Action based in Bangalore, India. All interviewees provided full informed consent. Transcripts and all quotes were fully anonymised.

## Results

Based on the analysis of the interviews, the following three perspectives on accountability were identified. At one end of the spectrum, we identified what we have called ‘accountability as targets’ (henceforth ‘Targets’) whereas at the other end we identified what we have called ‘accountability as transformation’ (henceforth ‘Transformation’), ‘accountability as efficiency’ (henceforth ‘Efficiency’) was identified in between.

### Accountability as Targets

In essence the ‘Targets’ perspective considers being accountable as meaning the accomplishment of targets for service coverage. These targets are based on expert-defined ‘needs’ of the community,

“*In service provision, we decide what the beneficiary wants, the beneficiary doesn’t decide what he or she wants.*” (IDI – 2, Govt. Official)

“*Because almost all the efforts were top-down… we saw accountability from the monitoring or the government accountability point of view. …find out how many were done… and the community was hardly a part of that process…*” (IDI – 1 Govt. Official)

Given the importance attributed to the reaching of pre-specified targets, a strong management information system was seen as a key component of the health system. Therefore, a gap between the community and the system was defined as a gap in information regarding coverage more than anything else. It was felt that, given the emphasis on reaching targets, the system would stop at nothing to achieve these targets,

“*Obviously the government would want to get it done… in the back of our mind… getting it done becomes much more important than many other things…*” (IDI – 1 Govt. Official)

Further, it was assumed that the mere consumption of services was adequate for the achievement of health. The importance of the social determinants and other contextual factors contributing to health were not emphasised.

From the perspective of targets, training was seen as a process of awareness building. The idea is that the people have to ‘know’ what services are available in the government health sector. It was assumed that knowing will automatically lead to individuals utilising these services. The persons who hold this perspective held that the training of key individuals in the community would spread health messages and the importance of consuming government-provided services for health. This, in turn, would increase the cooperation of the community and lead to the achievement of the targets.

Community-based monitoring was seen as an irritant and described as “*people stepping on our toes*”.

“*So that kind of a system is superfluous, rather I will put it as superfluous from one angle, one angle it is an irritant to the others. The same thing we are reviewing and doing the weekly review, monthly review and so many other reviews. When again they were coming in from different directions, the person who is sitting at the helm of affairs, he gets annoyed.*” (IDI – 3 Govt. Official)

The ‘Targets’ perspective held that the fact that the front-line workers were so well recognised, and that services were consumed by such a large proportion of the population, reflected the lack of a gap between the community and the system. In such a situation, external agencies were perceived as irritants. The entry of civil society organisations and NGOs into the implementation was not welcomed. Their inclusion was seen as contributing to the break in relationship between the community and the health system. Further, some of the interviewees even questioned the legitimacy of NGOs to implement these programmes despite the NRHM guidelines.

“*So if the suggestions were from the people rather than from the NGO, then the government would have accepted it. But the problem here, everything was from the NGOs…*” (IDI – 2 Govt. Official)

One of the issues that was raised by those who held the ‘Targets’ perspective was their concern that accountability to the community was being demanded precisely after decades of fiscal controls and interference that led to a decreased capacity and the erosion of motivation, especially among the front-line staff. Referring to the decline in postnatal services discussed in the interview, it was noted that,

“*The problem with postnatal care is, gradually we have withdrawn, knowingly or unknowingly the services at the sub-centre. That is the main reason where we have failed to have a postnatal care.*” (IDI- 3 Govt. Official)

This participant essentially made the point that the reasons for the lack of services were systemic, and many a time out of control of the public health department. Thus, it would not be appropriate for the communities to hold frontline health workers responsible for the gaps under these circumstances.

### Accountability as Efficiency

The ‘Efficiency’ perspective also saw accountability as being about the reaching of technically set targets. Like the ‘Targets’ perspective, these are also set by experts. However, the difference between this and the ‘Target’ perspective was that there was a recognition of the need for community feedback.

“*…let us ask people – what is the need they want and make it as our mandate… 100% we know what they want and we are doing all these things.*” (IDI – 4 Govt. Official)

There was an element of moving away from strictly expert-set targets to what may be termed the ‘community needs assessment’ approach. Yet, the overall content and priorities of the services were still to be set by the system. Further, there was also an acknowledgement of the importance of moving from outputs to outcomes.

“*See we have to have the capability to tell him that ‘see you are sick’, and we should have the capability to treat him and tell him that, ‘now you are okay’. And then go back to the community to do his job… I feel if we have such a system, then that is an accountable system. That is what I feel.*” (IDI – 4 Govt. Official)

Given the overarching focus on the management component, there remained a focus on quantitative targets; nevertheless, this perspective allowed for the concept of triangulation, introduced by the NRHM, for the framing of accountability as the correlation between multiple sources of information, with community-based monitoring as a way of cross-checking information available through the routine health system.

“*One good thing was this triangulation of accountability, in a sense, okay you do one thing, we would go to the community, we will also ask* [them what they felt] *and finally triangulate it. And that is one good aspect of it. Everywhere, the triangulation of data, work or whatever it is, evaluation, I think that is the ideal thing to be done.*” (IDI – 2 Govt. Official)

This perspective acknowledged a gap between the community and the system but seems to put the onus of change on the community to make full use of the programmes by demanding improved implementation. Further, it remains silent about the ways in which the system itself could be strengthened from within.

According to this perspective, training went beyond awareness building and should aim to build the community’s capacity. People holding this perspective believed that the community needs training and capacity-building in order to understand the logic and rationale behind the various services being provided by the system. This training and capacity-building was to be done by external agents (as they will do so more efficiently and flexibly). The community was expected at the end of such training to make demands that are “*more in line with what the department is providing*”, thus increasingly sharing or internalising the logic and rationale of the experts.

“*…so here one of the things we were also doing was moving the community up… to talk to the health system on an equal level and if not on an equal level somewhere near an equal level…*” (IDI – 1 Govt. Official)

“*The community will become more aware about what they can ask for from the health system and also more aware of their responsibilities*.” (IDI – 1 Govt. Official)

Community-based monitoring was seen as providing important information about the actual service delivery, including reach and quality.

“*With my experience I am saying that it is still a top-down approach and the system does something. And during the implementation process, there are some problems… because of the need of the community… now the system thinks back saying - how to handle the problem?*” (IDI – 4 Govt. Official)

External agencies like NGOs were seen as playing a crucial part in implementation, especially of non-routine programmes like those trying to implement community-based accountability. Left only to the government, such processes would never have even been implemented. This was attributed to the fact that the NGOs have more flexibility in functioning than government.

“*…and definitely NGOs supporting this programme is not a problem… if you introduce any new programme, there should be a momentum at the field level to actually carry out the work. With the current system structure, and with lots of mandates lined up in each table, I personally feel that the system will not have enough capacity in terms of human resources, time, and of course money is there; so time and other logistics to implement the programme. So, the system needs an extra push to actually roll-out the programme at ground level.*” (IDI – 4 Govt. Official)

Those holding this perspective did not explicitly mention the fiscal strain or interference in the functioning of the public health system, but seemed to take the lack of capacity of the public health system as a given and requiring “*external support*”.

### Accountability as Transformation

According to this perspective, the key feature of an accountable system was not only meeting the needs of the community, but also their aspirations, thus clearly expecting the community to play a greater role in defining the objectives of the system than the earlier two perspectives. Those holding this perspective felt that the services should be provided in such a way that the various social determinants, such as caste, class and gender, which are key axes along which inequity arises, are taken into account. They thus went beyond just the provision of services, and expected an accountable system to engage with the larger structural determinants of health. This was thus a radically different conceptualisation than the earlier two.

“*Accountability… is taking into account the present economic and social conditions… are the mechanisms taking into account these conditions?… the health system cannot destroy caste… but issues like caste… and the inequities that arise as a result of this… is the system able to address these or not?*” (IDI – 5 NGO leader)

This perspective saw the whole process as going beyond consultative processes for programme design and saw the ultimate accountable health system as one in which policy-makers, service providers and the community all sat down and discussed things as equals. In this situation, the community was an equal partner as a consequence of ‘citizenship’ and ‘rights’. Thus, this perspective critiqued the experts and the managers for ignoring key dimensions of various services or interventions that were important from the community point of view.

“*… for a person with TB, he has to have tablets thrice a week… he has to have DOTS* [directly observed treatment short course]*… The doctor would have seen it purely as a technical or a medical issue. But the community would not have seen it in that way… they would have seen it is a rights issue… it should be available… it is on this basis that the issue is seen.*” (IDI – 6 NGO leader)

Thus, accountability programmes were all about altering the power balance in favour of the community, and enabling the community to gain in confidence while interacting with the authorities and thus essentially deepening the democratic process.

“*… now, even if there is no project the doctor listens to me and talks to me… he sees me in a good light… see he has developed such confidence… this confidence is not only within the health system, but for the entire system in which he works… he enforces it…*” (IDI – 6 NGO leader)

The gap between the system and the community was thus one of power – at one level, it was the imposition of the biomedical understanding on to what is described as the ‘social understanding’ of life in the community, and at another level it was about who had the voice and power to plan programmes and determine the rules for the distribution of resources.

At the heart of the process, transformation is seen both at the structural level but equally importantly at the individual level. A number of examples of such transformation are given to highlight the point.

“*… it was teaching learning… it was thought provoking… those who were suppressed till now are beginning to think in a different way. Those who have never thought of such things are now thinking about them…* [referring to organising and demanding change]” (IDI – 6 NGO leader)

Training was thus an absolutely crucial aspect of any programme for those who held this perspective. It was about building up a feeling of citizenship and recognising that, as groups of citizens, they had a right to demand accountability from the system. More importantly, the training had to be sensitive to the unique socioeconomic, political and cultural context in which the particular programme was being implemented. In other words, the training is about laying bare the larger structural issues and demanding or creating an awareness and demand for structural change. This concept of training goes beyond ‘awareness’ and ‘capacity-building’ in the earlier two perspectives to the concept of ‘structural competence’.

“*… the third thing is to have trainings that will enable the reduction of all the obstacles that were envisaged and identified* [due to the socioeconomic and political structures]…” (IDI – 5 NGO leader)

Community-based monitoring was thus more than information gathering alone, and according to this perspective the process of monitoring was as important as the actual information collected. Firstly, knowledge about their entitlements was in itself empowering. Secondly, the information collected by the members and then collated into a report card greatly increased their negotiation power in any discussion with the health system, altering the power balance in favour of the people.

“*… in this process everyone got a chance, an opportunity to sit down and discuss… this was a key point… I saw the discussion as the crucial point… it was not only the doctor and nurse who dominated the space… anyone, a lay person, an ill person, a technical person, a non-technical person, a member of a particular group… anyone could enter and take part in this space.*” (IDI – 6 NGO leader)

This perspective saw accountability as the building up of more equal relationships between the system and the communities they serve. The key issue was the power hierarchy. In such an unequal situation it is crucial for there to be an external agency to help the community negotiate these power differences as they gradually gained in confidence and were empowered in the process. The external agencies were not only to conduct the training and capacity-building of the community but were also crucial in acting as a balance between the system and the community.

“*… because these obstacles have been around for so long and we have not been able to make things better so… then external help is required… that is all… definitely there is the need for someone in a supportive role…*” (IDI – 5 NGO leader)

“*… so if the public health department takes everything in its hand … the process will de facto collapse … there is no countervailing power…*” (IDI – 5 NGO leader)

## Discussion

Our research establishes the presence of three distinct ways in which the concept of accountability has been framed by key actors (within the Department of Health and among the civil society organisations in the state) involved in the formulation and implementation of policy aiming to strengthen community-based accountability of the healthcare system in Tamil Nadu, India. The research also reveals how these different framings imply quite different conceptualisations of the role of the healthcare system as well as expected outcomes of a programme aimed at increasing the accountability of the health system. It also means that the roles of different actors and the components of such a programme are seen differently.

It is important to recognise the rapidly changing relationships between public services and citizens as we study accountability.

An increasing dominance of neo-liberal ways of thinking about the state has been noted [21]. This is leading to an increasing fragmentation of the production, provision, financing and governing of these services. In such circumstances the lines of accountability and the ability to ascribe responsibility are becoming increasingly complex. Further, under such circumstances, traditional forms and notions of accountability are inadequate to the task of dealing with this complexity [21].

The relevance of the presence of these differing perspectives needs to be placed in the context of the post-colonial setting. It is well established that the evolution of the state, the role it has played in the development of newly independent nations, and the subsequent impact of the changing macroeconomic situation is different from the way these processes took place in Western ‘developed’ nations. In India, in particular, it has been pointed out that the political elite chose to follow a path of bureaucracy-driven development rather than a mobilisational approach [28]. This, combined with the ideology of expert-driven planning as the pathway for the country’s development, meant that people were seen as passive recipients of the benefits of science and technology and had to give up their ‘ignorant’ and ‘traditional’ ways. This obviously had significant impact on the way the concept of accountability was translated into programmes and practices and how it was interpreted in the various institutions of government. Studies of accountability in health systems in India point out, for example, that the poor are more often seen as supplicants rather than rights holders [8] and their marginalised status obstructs their ability to make claims in the first place [8, 29]. Research from the systems perspective points out that accountability of peripheral health workers is more towards their superiors (in highly hierarchical health systems) rather than to the needs of the people they serve [30], and that hierarchical structures mean that accountability processes invariably seek a scape goat rather than systemic change [31]. This illustrates the way in which historic, structural and social aspects of institutions in a particular setting play a key role in the way accountability initiatives are implemented and experienced. It is in this context that the presence of three distinct perspectives is significant.

Given these findings, one way of interpreting these three distinct perspectives of accountability is to relate them to the changing underlying modes of governance. Thus, the ‘Targets’ perspective can be seen to reflect the mode of governance of post-colonial states variously characterised as ‘highly modern’ [32] or ‘interventionist’ [28]. This mode of governance is heavily expert and science driven, and bureaucratises approaches to development [28]. The ‘Efficiency’ perspective could be placed within the ‘New Public Management’ framework of governance, in which systems are still expert driven in terms of content and prioritisation, and states increasingly constrained. In this perspective, public systems draw on resources from communities, civil society and markets for the provision of services. Both of these can be contrasted to the ‘Transformative’ perspective, which may be termed more radical and transgressive [10] and in which the accountability-related processes rest on the foundation of explicit recognition of and attempts to reduce power differentials between communities and the state.

Viewed from the perspective of the way the community and its role is conceptualised in these frames, it can be seen that these three groupings also almost completely map on to the categorisation of accountability made by Murthy and Klugman when they referred to “*lower*”, “*middle*” and “*higher*” degrees of “*accountability to communities*” [33]. As described by the authors, there is a need for a “*higher degree of accountability signifying accountability of not only health workers but also policy makers, to public as well as higher up, with respect to issues of choice of services as well as delivery of inputs, to both prevent and detect errors*” [33]. This also reflects the way the community is seen in differing modes of governance.

Further underlining this difference are the obvious tensions between the perspectives – especially among the actors within the public health system. There was a group who sharply resisted the imposition of these top-down concepts of accountability to communities by an administration seen as “*not able to understand ground reality*” and that was instrumental in eroding the health system’s capacity in providing services and its overall autonomy in planning. This was contrasted by the other officials who took the limited state capacity as a given and thus saw a natural role for the external agencies to ensure the effectiveness and reach of the state.

Yet another way in which we can see these perspectives is that they are all demanding accountability, but at different points in the implementation process and with regards to different dimensions [7]. The ‘Targets’ and ‘Efficiency’ perspective take an institutional perspective focusing on organisational goals, procedures and guidelines and hierarchical implementation systems. The ‘Transformation’ perspective clearly focuses on the relationship between system and community and on the social dimensions emphasising equity and fairness. Therefore, from the implementation point of view, one finds agreement on particular interventions (community-based monitoring in the case discussed) at a superficial functional level, but at the same time conflicting underlying framings, which ultimately lead to abrupt termination [20]. Thus, the issue at hand is not that these are conflicting frames of accountability but that they all focus on different dimensions. Theoretically, all these dimensions of accountability are crucial for any comprehensive conceptualisation or programmatic formulation of accountability or community-based accountability. However, in our opinion, their existence points to deeper underlying beliefs that need to be explored.

While there is an increasing number of calls for accountability from civil society as well as government, it is important to discern who is demanding this accountability and with what framing of the issue. Given that the underlying beliefs and the way the problem is framed is crucial to programme design, these different framings have clear impact on the way programmes are designed. What is critical to focus on is the way they function in opening up particular subject positions in a given situation. Thus, while in the ‘Targets’ and ‘Efficiency’ perspectives the citizen may only ask or demand for the availability of an entitled service or indeed demand better quality, they may not (unlike in the ‘Transformation’ perspective) demand a change in the designs or content of various programmes. This obviously has significant implications for the way in which programmes are designed and institutions of accountability are structured.

The three perspectives that are emergent from the interviews clearly demonstrate that there are deep differences among the perspectives that likely arise from an ontological level. These deep differences, as demonstrated above, have implications for the meaning or importance given to various components of any programme that is planned. Therefore, while there may be some consensus at the policy formulation level, the way the policy components are interpreted at each level of the implementation process depends on the perspective that is dominant at that level, thus predicting a messy and complex process or policy reinterpretation at each level. This is highlighted by the differing views of the people espousing the different frames on various design aspects of any accountability programme, including training, monitoring and the role of an external agency.

### Limitations

This paper is based on interviews with individuals who were actively involved in the implementation of the community-based accountability components of the NRHM in the state of Tamil Nadu. We had to be sensitive to the nature of the hierarchy of the health system and limit our description of who we interviewed in order to maintain anonymity. Moreover, the main intention of this paper is to highlight and attempt to delineate the presence of distinct perspectives on accountability present among the key actors of the implementation of the CAH programme. The attempt was not to define the composition of the groups exhaustively. Thus, we have refrained from drawing conclusions as to the composition or the extent of support of the groups, and merely defined the differing perspectives. While the small numbers may be seen as a shortcoming of the study, given the particular setting of Tamil Nadu as well as the type of conclusion we draw in this paper, we feel we have done justice in the circumstances. This paper was later shared with some of the key informants who endorsed the interpretations and the conclusions reached.

## Conclusions

The finding of three distinct perspectives emphasising (1) the achievement of institutional targets (Accountability as Targets), (2) increasing the efficiency of services with community or civil society inputs (Accountability as Efficiency), and (3) radically altering power differentials between the system and community (Accountability as Transformation), gains particular significance in the context of increasing calls for accountability of government and public systems in developing country settings. Here, the production and delivery of these services are increasingly moving from public to private players. The relationship of public systems with those they serve, especially the marginalised communities, takes on particular forms in post-colonial settings.

While the historical development of public systems in post-colonial settings involved a very paternalistic state, changing norms of governance due to the pressure from international financial institutions opens up spaces for the emergence of newer modes of engagement with citizens. This opening of spaces, we argue, is what is reflected in the presence of three distinct arguments in the policy subsystem dealing with accountability mechanisms in the state of Tamil Nadu.

While this opening up of spaces enabled previously unexplored ideas to come to the fore thanks to the presence of civil society representatives at the drawing table, the lack of mechanisms of institutionalised policy learning in a hierarchical bureaucratic environment means that such ideas are merely thrust upon the system in a top-down fashion. These are rarely sustained once the opportunity structures that enabled these ideas to come onto the policy agenda in the first place change. Thus, while it is important to see these spaces and opportunities as dynamic and contingent, and therefore as spaces for the ongoing struggle, it is equally important to call for the evolution of institutional mechanisms for this engagement, using newer ideas that are open and transparent and that go beyond the purely hierarchical and top-down mode of dealing with policy change.

As has been noted above, it has been suggested that it may be useful to see these differing perspectives not as conflicting but as merely referring to different aspects of the implementation process as discussed above [7]. It is tempting to suggest that all one needs is a fusing of these together. Our research, however, reveals that the differing focus of the different framings rises from radically different underlying assumptions. Further, taking into account the demands of equity and justice put forth by the ‘Transformation’ perspective requires system level changes and not merely tinkering at the level of project design. This is revealed by both the link between poor performance and systemic underfunding due to macroeconomic change pointed out in the interviews, and the underlying complexity of public system institutional arrangements referred to above.

While there may be a larger consensus with regards to the need for accountability of public systems in general, and health systems in particular, to the communities they serve, this macrolevel consensus is not enough to ensure the implementation of these concepts, but in fact needs to contend with deep-seated diversity in perspectives. From this, it can be concluded that long-term sustained transformation of public systems into truly accountable systems in the sense used by the ‘Transformative’ perspective has to happen in sync with larger forces and movements that work at the level of norms and beliefs, and institutional mechanisms to engage with this diversity rather than only focusing on policy or resources.

## Abbreviations

CAH: Community Action for Health
NGO: non-governmental organisation
NRHM: National Rural Health Mission
